# Supplementary material for: Association of variations in HLA class II and other loci with susceptibility to EGFR-mutated lung adenocarcinoma
Source: Nat Commun. 2016 Aug 9;7:12451. doi: 10.1038/ncomms12451 (PMC4980483; doi:10.1038/ncomms12451)
Supplement: Supplementary Figures and Supplementary Tables — Supplementary Figures 1-3 and Supplementary Tables 1-17 [file ncomms12451-s1.pdf]

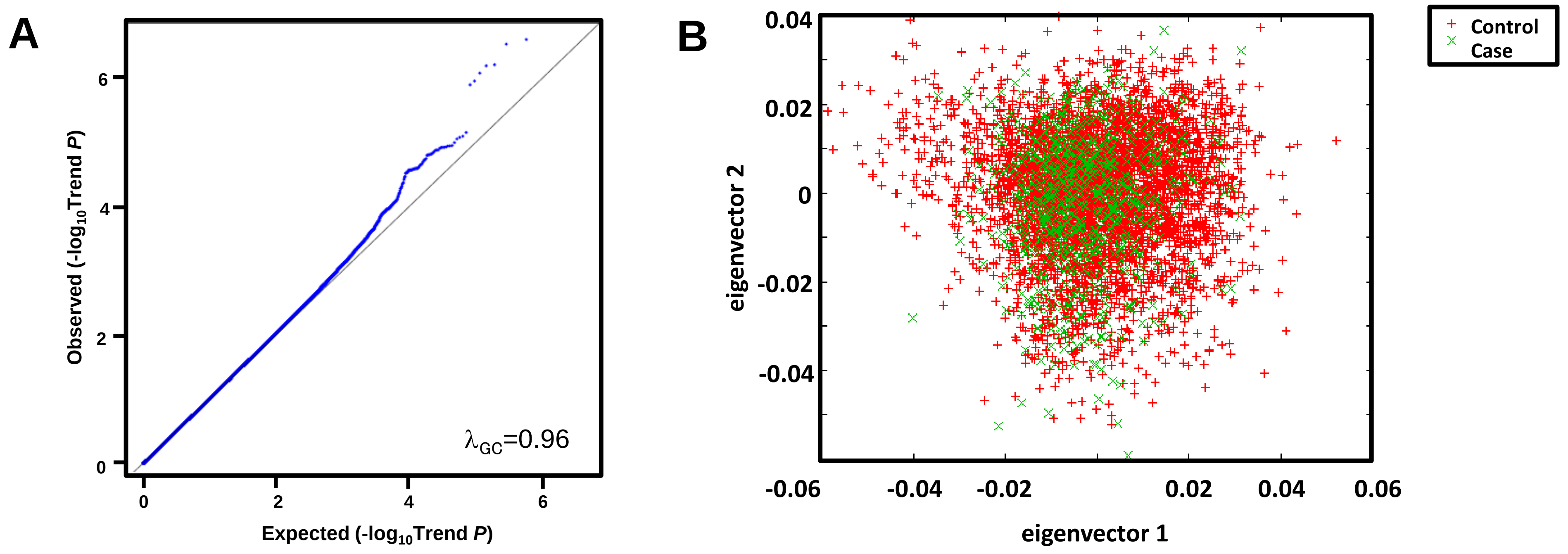

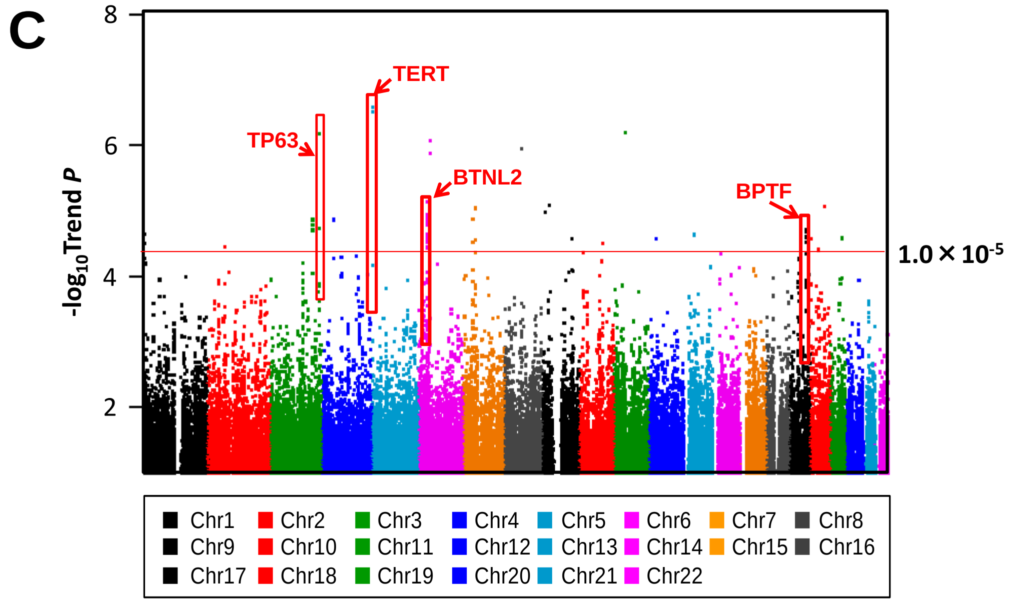

Supplementary Fig. 1. Representations of quality control performed in the GWAS

A. Quantile-quantile (Q-Q) P_{Trend} value plot for the test statistics (logistic regression trend tests) in the GWAS. Q-Q plots of the observed *P*-values ($-\log_{10}P_{\mathrm{Trend}}$) for cases typed on the Illumina Omni1-Quad beadChip and controls typed on the Illumina OmniExpress beadChip are shown. The genomic inflation factor (λ_{GC}) was 0.96. B. Both cases and controls in the study belonged to same cluster according to PCA. C. Manhattan plot of GWAS of lung adenocarcinoma with the exon 19 in-flame deletion mutation or the L858R exon 21 mutations in *EGFR* in the Japanese population. The plot is based on a logistic regression model adjusted for age, gender, and smoking status. Four known loci described in the text are marked by boxes.

(1) GWAS

Control: HumanOmniExpress

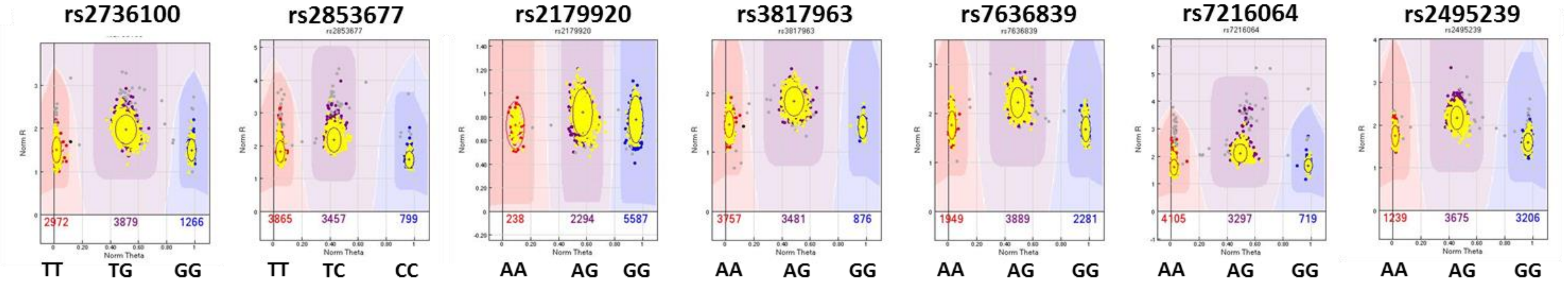

Case: HumanOmni1-Quad

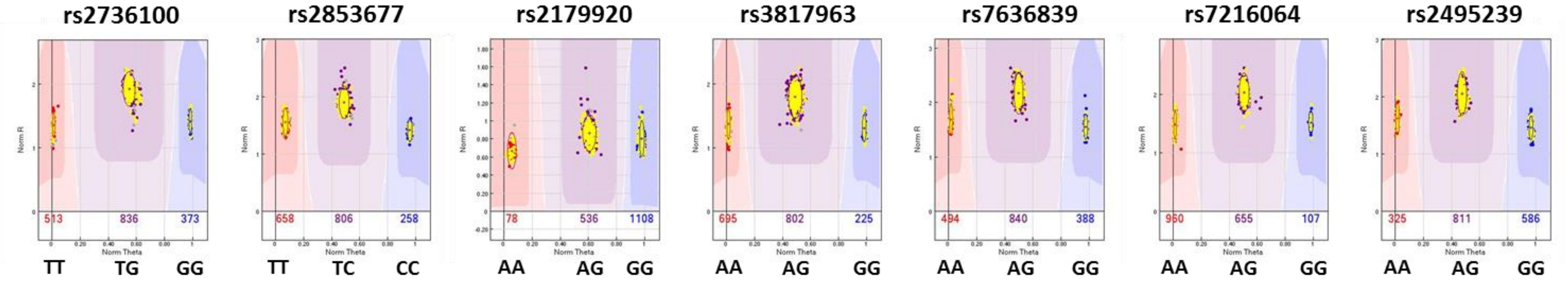

(2) Validation set 1

Control: HumanOmniExpress

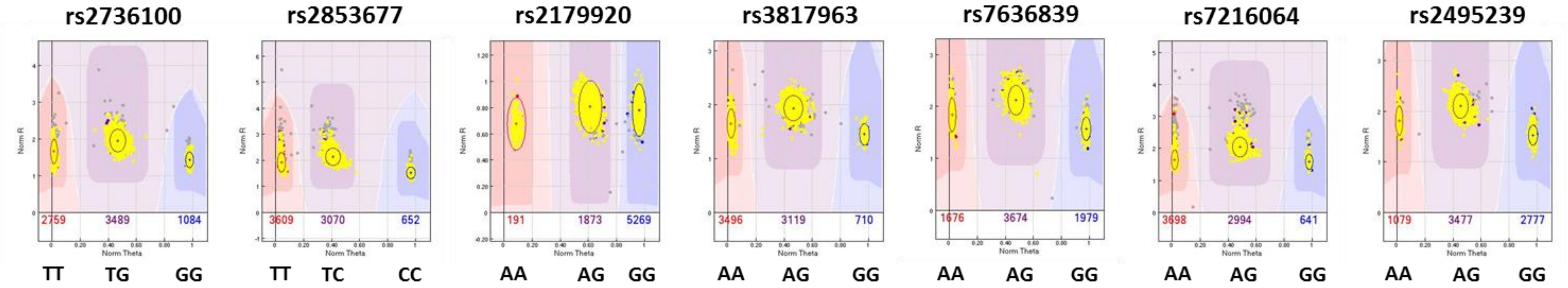

Supplementary. Fig. 2 (*continued*)

(2) Validation set 1

Case: invader assay: rs no. (allele1/2)

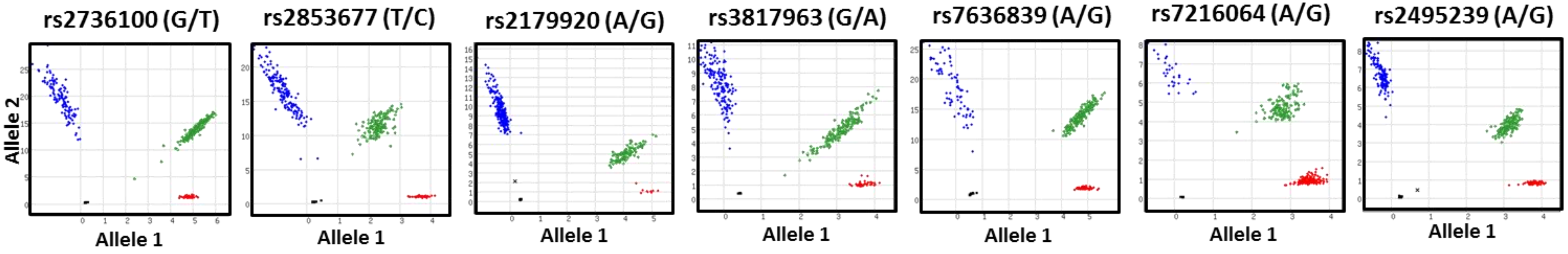

(2) Validation set 2

Control: HumanOmni2.5-8: rs no. (allele1/2)

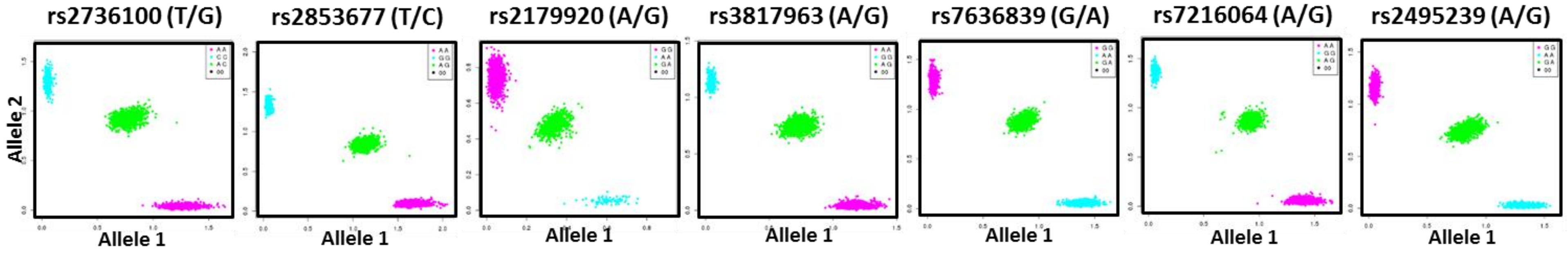

Case: TaqMan assay

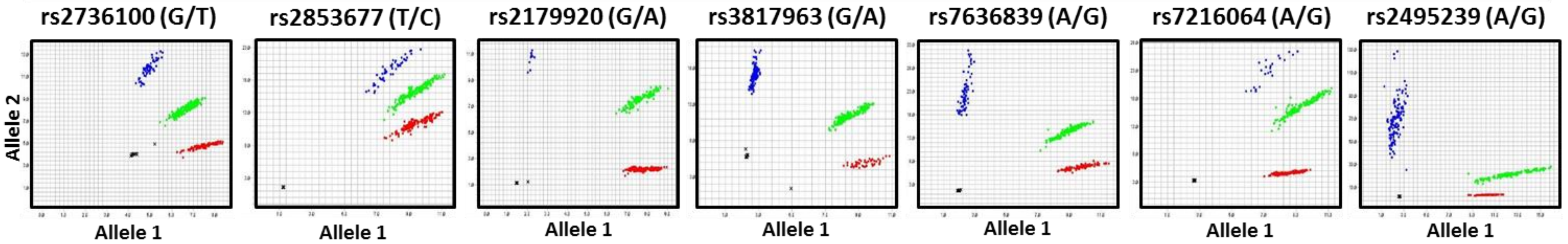

Supplementary Fig. 2. Cluster plotting for the seven SNPs that attained significance at Trend $P < 5.0 \times 10^{-8}$ in the combined analysis

Subjects of GWAS and validation set 1 shows yellow plots.

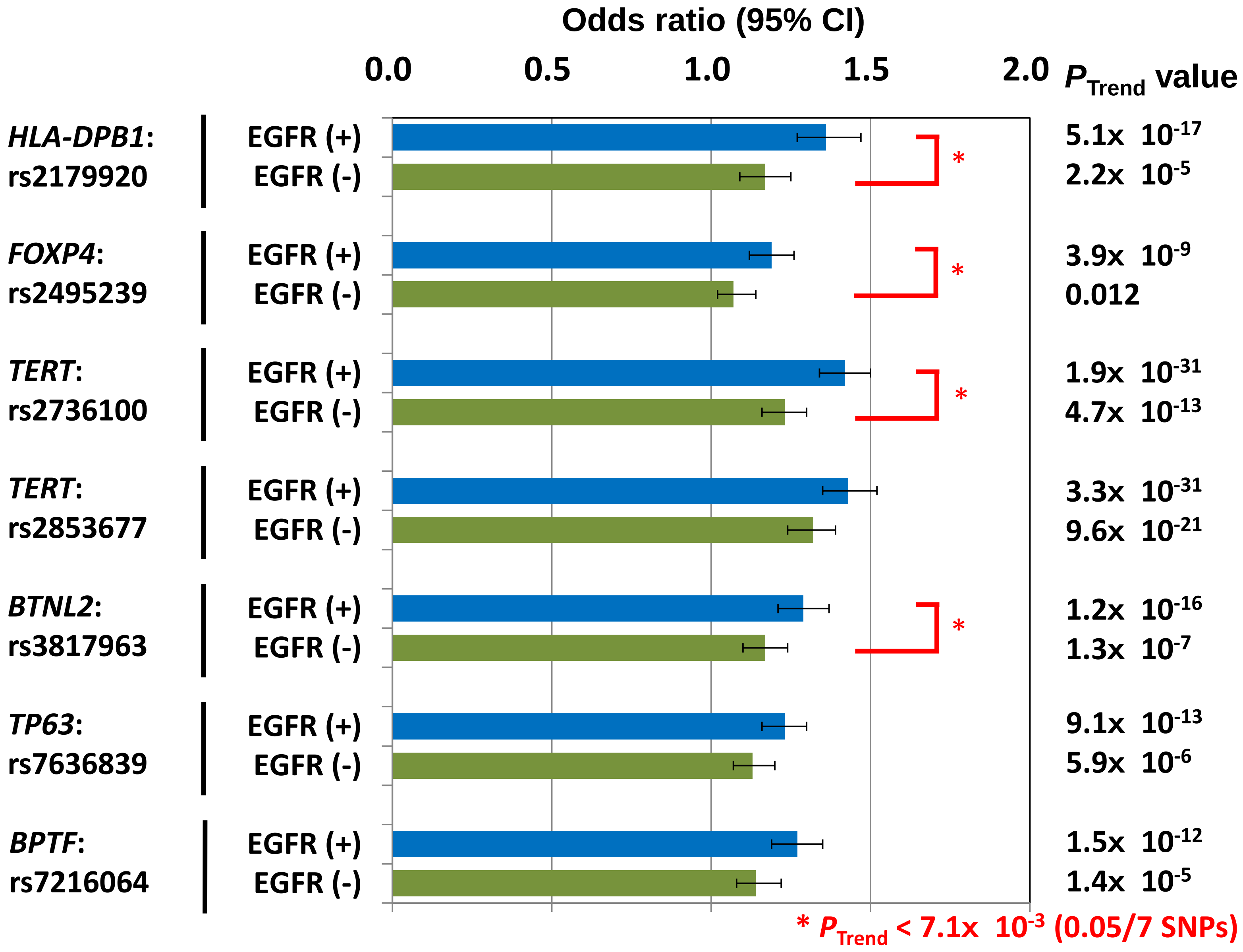

Supplementary Fig. 3. Association of 7 SNPs with risk for lung adenocarcinoma according to somatic *EGFR* mutation

Case-control analysis; ORs of all seven SNPs are higher for LADC with *EGFR* mutation than without. Case-case analysis; four of the seven SNPs (marked in RED*) show statistically significant allelic differentiation between LADC cases with and without *EGFR* mutation. The trend *P* was calculated by a logistic regression analysis.

Supplementary Table 1. Cases and controls analyzed in the present study

Subject	Lung adenocarcinoma cases		Control
	Lung adenocarcinoma with *EGFR* mutation[a,d]	without *EGFR* mutation[a]	
Total	3,173	3,694	15,158
Female (%)	1,962 (61.8)	1,314 (35.6)	6,628 (43.7)
Age, yr (mean)	64.5	63.7	47.7
Never-smoker (%)	1,955 (61.6)	1,179 (31.9)	7,578 (50.0)
GWAS			
Source	NCCH[b]	NCCH[b]	BioBank Japan
Genotyping platform	HumanOmni1-Quad	HumanOmni1-Quad	HumanOmniExpress
Number of samples	663	631	4,367
Female (%)	454 (68.5)	274 (43.4)	1681 (38.5)
Age, yr (mean ± SD)	59.8 ± 7.9	59.0 + 9.1	60.3 ± 11.6
Never-smoker (%)	458 (69.1)	280 (44.4)	1676 (38.4)
Validation set 1			
Source	NCCH[b] and Kanagawa Cancer Center.	NCCH[b] and Kanagawa Cancer Center.	BioBank Japan
Genotyping platform	Invader assay	Invader assay	HumanOmniExpress
Number of samples	1,275 (1,056 and 219, respectively)	1748 (1,631 and 117)	6,817
Female (%)	706 (55.4)	542 (31.0)	3198 (46.9)
Age, yr (mean ± SD)	64.8 ± 10.1	62.7 ± 10.6	44.6 ± 18.1
Never-smoker (%)	677 (53.1)	426 (24.4)	3503 (51.4)
Validation set 2			
Source	NCCH[b], Akita Univ. Hospital and Gunma Univ Hospital.	NCCH[b], Akita Univ Hospital. and Gunma Univ. Hospital.	JPDSC[c], NCCH[b] and Keio Univ.
Genotyping platform	TaqMan assay	TaqMan assay	HumanOmni2.5 and TaqMan assay
Number of samples	1,235 (900, 136 and 199, respectively)	1,315 (886, 207 and 224, respectively)	3,974 (2,823, 368 and 783, respectively)
Female (%)	802 (64.9)	498 (37.9)	1,749 (44.0)
Age, yr (mean ± SD)	66.8 ± 9.5	67.3 ± 10.2	39.1 ± 13.1
Never-smoker (%)	820 (66.4)	473 (36.0)	2,399 (60.4)

[a]EGFR somatic mutations were tested in lung adenocarcinoma patients by HRM analysis, invader assay, ARMS or PNA-LNA PCR clamp method. [b]National Cancer Center Hospital. [c]Japan PGx Data Science Consortium. [d]The genetic association data were deposited in GWAS central.

Supplementary Table 2. Overlaps of the case and control subjects/data with those in our previous GWAS

Category	Lung adenocarcinoma cases		Control
	with EGFR mutation	WITHOUT EGFR mutation	
GWAS	663 cases: genotype data from a previous GWAS* was used	631 cases: genotype data from a previous GWAS* was used	4,367 controls: genotype data from a previous GWAS* was used
Validation set 1	308 cases: analyzed in a previous GWAS*	826 cases: analyzed in a previous GWAS*	6,817 controls: genotype data from a previous GWAS* was used
	967 cases: newly analyzed	922 cases: newly analyzed	
Validation set 2	262 cases: analyzed in a previous GWAS*	323 cases: analyzed in a previous GWAS*	1,151 controls: analyzed in a previous GWAS*
	973 cases: newly analyzed	992 cases: newly analyzed	2,823 controls: newly analyzed

*Shiraishi et al., Nat Genet. 2012; 44(8): 900-903.

Supplementary Table 3. Associations of previous susceptibility loci with risk for lung adenocarcinoma with *EGFR* mutation

SNP ID	Location	Chr	Position (hg38)	Gene	Allele		Allele frequency[a]				SNP in LD	R^2	Frequency[d]		OR	(95% CI)	P_{Trend} value[e]	Reference
					Ref	Alt	AFR	AMR	EAS	EUR			Case	Ctrl				
rs4488809	3q28	3	189,638,472	*TP63*	T	C	0.34	0.38	0.52	0.49	rs7636839	0.99[b]	0.457	0.521	0.78	(0.68-0.87)	1.80E-05	Miki, 2010., Shiraishi, 2012.
rs10937405	3q28	3	189,665,394	*TP63*	C	T	0.31	0.31	0.33	0.43	-	-	0.269	0.323	0.78	(0.68-0.89)	2.31E-04	Miki, 2010., Wang, 2015.
rs2736100	5p15.33	5	1,286,401	*TERT*	A	C	0.43	0.45	0.39	0.50	-	-	0.468	0.391	1.37	(1.22-1.42)	2.51E-07	Miki, 2010., Landi, 2009., Shiraishi, 2012., Wang, 2015.
rs31489	5p15.33	5	1,342,599	*CLPTM1L*	C	A	0.48	0.31	0.16	0.43	-	-	0.146	0.143	1.03	(0.87-1.22)	0.73	Landi, 2009.
rs3817963	6p21.3	6	32,400,310	*BTNL2*	T	C	0.11	0.31	0.23	0.27			0.388	0.327	1.30	(1.15-1.45)	2.33E-05	Shiraishi, 2012.
rs13194504	6p22.1	6	28,662,914	-	G	A	0.00	0.02	0.00	0.07	-	-	-	-	-	-	-	Landi, 2009.
rs4324798	6p22.1	6	28,808,340	*NOP56P1 - RPL13P*	G	A	0.09	0.03	0.00	0.07	-	-	-	-	-	-	-	Landi, 2009.
rs3749971	6p22.1	6	29,374,998	*OR12D3*	G	A	0.02	0.02	0.03	0.08	-	-	0.102	0.089	1.21	(0.99-1.48)	0.062	Landi, 2009.
rs3117582	6p21.3	6	31,652,743	*BAG6*	T	G	0.04	0.20	0.00	0.07	-	-	-	-	-	-	-	Landi, 2009.
rs2395185	6p21.32	6	32,465,390	HLA Class II region	G	T	0.14	0.36	0.34	0.32			0.412	0.380	1.17	(1.03-1.32)	0.014	Lan, 2012.
rs9387478	6q22.2	6	117,465,017	*ROS1* , *DCBLD1*	C	A	0.86	0.47	0.48	0.52	rs9372480	0.860[b]	0.449	0.481	0.90	(0.80-1.01)	0.084	Lan, 2012.
rs7086803	10q25.2	10	112,738,717	*VTI1A*	G	A	0.60	0.12	0.30	0.03	rs1885281	1.000[b]	0.246	0.231	1.09	(0.95-1.26)	0.20	Lan, 2012.
rs2036534	15q25.1	15	78,534,606	*AGPHD1*	T	C	0.23	0.45	0.44	0.23	rs7163730	1.000[c]	0.492	0.473	1.04	(0.93-1.08)	0.54	Landi, 2009.
rs1051730	15q25.1	15	78,601,997	*CHRNA3*	G	A	0.09	0.25	0.03	0.36	-	-	0.025	0.025	0.96	(0.71-1.16)	0.83	Landi, 2009.
rs938682	15q25.1	15	78,604,205	*CHRNA3*	G	A	0.33	0.49	0.45	0.23	rs6495308	1.000[c]	0.204	0.220	0.90	(0.78-1.05)	0.18	Landi, 2009.
rs12914385	15q25.1	15	78,606,381	*CHRNA3*	C	T	0.20	0.27	0.32	0.40	-	-	0.305	0.308	0.97	(0.91-1.08)	0.65	Landi, 2009.
rs7216064	17q24.3	17	67,902,693	*BPTF*	A	G	0.17	0.26	0.65	0.22			0.759	0.706	1.35	(1.18-1.55)	2.05E-05	Shiraishi, 2012.

Chr; Chromosome, OR; odds ratio, CI; confidence interval, Ref; Reference, Alt; Alteration. AFR; African, AMR; Admixed American, EAS; East Asian; and EUR; European.

[a]Allele frequency in 1000 genomes. [b]LD between SNPs determined in reference to 1000 ESA genomes. [c]LD between SNPs determined in reference to 1000 EUR genomes. [d]Allele frequency in the present GWAS cohorts. [e]Adjusted for age, gender and smoking status.

Supplementary Table 4. Association results of 43 SNPs that showed an association of $P < 10^{-4}$ in GWAS

SNP ID[a]	Chr	Position	Risk allele		GWAS (Cases 663 vs control 4,367)						Validation set 1 (Case 1,275 vs Control 6,817)					
			Ref	Alt	Risk allele frequency		P_{trend}[b]	OR	95% CI		Risk allele frequency		P_{Trend}[b]	OR	95% CI	
					Case	Ctrl			Lower	Upper	Case	Ctrl			Lower	Upper
rs4460663	1	7,186,325	C	T	0.578	0.519	6.23E-05	1.28	1.13	1.44	0.530	0.526	0.45	1.04	0.94	1.14
rs10915495	1	4,029,733	A	G	0.184	0.138	2.23E-05	1.41	1.20	1.65	0.137	0.133	0.86	1.01	0.88	1.16
rs13010493	2	67,123,397	A	C	0.926	0.888	3.46E-05	1.59	1.28	1.99	0.904	0.892	0.062	1.16	0.99	1.36
rs1320182	3	123,834,347	G	A	0.630	0.570	6.14E-05	1.28	1.14	1.45	0.587	0.587	0.95	1.00	0.91	1.10
rs7636839[c]	3	189,356,941	G	A	0.543	0.479	1.80E-05	1.29	1.15	1.46	0.520	0.478	1.09.E-04	1.20	1.10	1.32
rs3912608	3	162,654,424	A	G	0.115	0.078	1.33E-05	1.54	1.27	1.86	0.073	0.076	0.76	0.97	0.81	1.16
rs11939173	4	72,672,158	T	C	0.911	0.871	4.98E-05	1.53	1.24	1.87	0.858	0.864	0.28	0.93	0.81	1.06
rs11097821	4	75,534,357	C	A	0.534	0.474	8.88E-05	1.26	1.12	1.42	0.497	0.492	0.56	1.03	0.94	1.13
rs10857139	4	130,222,259	A	G	0.424	0.367	4.83E-05	1.29	1.14	1.46	0.353	0.364	0.56	0.97	0.88	1.07
rs4495020	4	174,920,657	G	A	0.192	0.154	9.27E-05	1.37	1.17	1.60	0.161	0.156	0.46	1.05	0.92	1.19
rs2736100[c]	5	1,286,516	T	G	0.468	0.391	2.51E-07	1.37	1.21	1.54	0.480	0.385	8.98E-18	1.53	1.39	1.68
rs2853677[c]	5	1,287,194	A	G	0.377	0.308	2.96E-07	1.38	1.22	1.56	0.387	0.298	1.44E-16	1.53	1.38	1.69
rs9277555	6	33,055,605	C	T	0.624	0.562	1.13E-05	1.32	1.17	1.50	0.596	0.578	0.13	1.08	0.98	1.19
rs3117221	6	33,061,947	G	A	0.623	0.560	7.05E-06	1.33	1.17	1.51	0.596	0.578	0.10	1.08	0.98	1.19
rs2477842	6	41,489,441	A	G	0.655	0.585	1.27E-06	1.36	1.20	1.53	0.610	0.600	0.57	1.03	0.93	1.13
rs7769173	6	11,224,748	G	T	0.466	0.414	8.86E-05	1.27	1.13	1.43	0.399	0.409	0.19	0.94	0.85	1.03
rs3817963[c]	6	32,368,087	T	C	0.388	0.327	2.33E-05	1.30	1.15	1.48	0.383	0.309	9.64E-11	1.38	1.25	1.53
rs2179920[c]	6	33,058,874	C	T	0.222	0.174	8.53E-05	1.34	1.16	1.55	0.204	0.154	2.12E-09	1.45	1.28	1.63
rs2495239[c]	6	41,490,488	G	A	0.448	0.374	8.40E-07	1.34	1.19	1.51	0.413	0.383	5.21.E-03	1.15	1.04	1.26
rs10951449	7	35,596,304	T	C	0.699	0.643	2.97E-05	1.32	1.16	1.50	0.636	0.653	0.34	0.95	0.86	1.05
rs6979267	7	35,605,025	A	G	0.649	0.589	1.33E-05	1.32	1.16	1.49	0.582	0.602	0.40	0.96	0.87	1.06
rs10238969	7	35,609,340	T	C	0.759	0.712	7.83E-05	1.32	1.15	1.51	0.702	0.725	0.35	0.95	0.86	1.06
rs2471552	7	45,977,173	C	T	0.825	0.775	8.90E-06	1.41	1.21	1.64	0.788	0.770	0.076	1.11	0.99	1.24
rs6988624	8	64,931,948	A	G	0.069	0.039	1.11E-06	1.88	1.46	2.42	0.044	0.047	0.55	0.93	0.74	1.17
rs10812169[c]	9	25,068,476	G	A	0.906	0.863	8.07E-06	1.58	1.29	1.93	0.881	0.871	0.033	1.17	1.01	1.35
rs2274419	9	113,538,122	A	G	0.800	0.747	7.74E-05	1.34	1.16	1.56	0.762	0.762	0.53	0.97	0.86	1.08
rs913080	9	116,555,563	G	A	0.529	0.475	8.25E-05	1.27	1.13	1.43	0.494	0.487	0.47	1.04	0.94	1.14
rs4740924	9	8,011,107	T	C	0.081	0.052	1.01E-05	1.67	1.33	2.10	0.051	0.054	0.11	0.84	0.68	1.04
rs1968028	10	90,662,086	G	A	0.633	0.572	3.04E-05	1.30	1.15	1.47	0.576	0.589	0.54	0.97	0.88	1.07
rs1907323	10	78,336,015	C	T	0.494	0.440	9.55E-05	1.27	1.13	1.44	0.451	0.438	0.40	1.04	0.95	1.14
rs3825165	12	26,555,730	A	G	0.235	0.191	2.60E-05	1.36	1.18	1.57	0.209	0.202	0.14	1.09	0.97	1.22
rs7156116	14	32,007,373	C	T	0.086	0.056	4.47E-05	1.58	1.27	1.96	0.068	0.059	0.10	1.17	0.97	1.42
rs740037	14	71,660,853	G	A	0.176	0.133	9.09E-05	1.38	1.17	1.61	0.149	0.145	0.39	1.06	0.93	1.21
rs8020912[c]	14	103,847,274	C	T	0.419	0.360	7.08E-05	1.27	1.13	1.44	0.403	0.372	7.97.E-03	1.14	1.03	1.25
rs8026785	15	50,632,662	C	T	0.729	0.672	7.58E-05	1.30	1.14	1.49	0.668	0.668	0.57	1.03	0.93	1.13
rs341392	15	61,124,235	C	A	0.465	0.404	9.69E-05	1.27	1.12	1.43	0.416	0.409	0.22	1.06	0.96	1.17
rs3910232	16	82,673,410	T	C	0.379	0.330	8.12E-05	1.28	1.13	1.45	0.332	0.330	0.77	1.02	0.92	1.12
rs4791114[c]	17	65,778,573	T	C	0.822	0.772	2.91E-05	1.39	1.19	1.61	0.800	0.780	7.77.E-03	1.17	1.04	1.32
rs7216064[c]	17	65,898,809	G	A	0.759	0.706	2.05E-05	1.35	1.18	1.55	0.741	0.708	5.54E-05	1.24	1.12	1.38
rs4545872	17	35,897,332	G	A	0.234	0.189	5.28E-05	1.35	1.17	1.55	0.193	0.202	0.29	0.94	0.83	1.06
rs1507727	18	1,438,095	C	A	0.808	0.753	2.59E-05	1.38	1.19	1.60	0.774	0.767	0.34	1.06	0.94	1.18
rs746172	18	32,751,792	C	T	0.253	0.205	3.83E-05	1.34	1.17	1.54	0.219	0.207	0.10	1.10	0.98	1.24
rs12455210	18	55,145,377	C	T	0.496	0.428	8.41E-06	1.31	1.17	1.48	0.413	0.444	6.04.E-03	0.88	0.80	0.96

[a]SNPs with $P_{\text{Trend}} < 10^{-4}$ in GWAS are shown. [b]Adjusted for age, gender and smoking status. [c]Ten SNPs showing ORs of $P_{\text{Trend}} < 0.05$ in the same direction in the 1st validation set.

Supplementary Table 5. Association results of 10 SNPs subjected to the second validation study

SNP ID[a]	Chr	Position	Allele		Validation set 2 (1,235 Cases vs 3,974 Controls)					
			Ref	Risk	Risk allele frequency		P_{Trend} value*	OR	95% CI	
					Case	Ctrl			Lower	Upper
rs7636839	3	189,356,941	G	A	0.530	0.480	1.62.E-05	1.22	1.12	1.34
rs2736100	5	1,286,516	T	G	0.461	0.386	3.64.E-10	1.35	1.23	1.48
rs2853677	5	1,287,194	T	C	0.383	0.309	5.78.E-11	1.38	1.26	1.53
rs2495239	6	41,490,488	G	A	0.422	0.387	4.72.E-03	1.15	1.04	1.26
rs3817963	6	32,368,087	A	G	0.356	0.317	2.90.E-04	1.20	1.09	1.32
rs2179920	6	33,058,874	G	A	0.214	0.174	5.56.E-06	1.30	1.16	1.46
rs10812169	9	25,068,476	G	A	0.865	0.871	0.26	0.93	0.81	1.06
rs8020912	14	103,847,274	C	T	0.391	0.383	0.55	1.03	0.94	1.13
rs7216064	17	65,898,809	G	A	0.749	0.703	4.34.E-05	1.24	1.12	1.38
rs4791114	17	65,778,573	T	C	0.803	0.774	4.72.E-03	1.18	1.05	1.32

*Adjusted for gender and smoking status.

Supplementary Table 6. Association of 7 SNPs with risk for lung adenocarcinoma with EGFR mutation according to gender and smoking status

SNP ID-risk allele (Locus)	Gene	Category	Number of Case*			Number of Control*			Risk allele frequency		P_{Trend} value	Crude OR (95%CI)
			AA	Aa	aa	AA	Aa	aa	Case	Control		
rs2736100-G	*TERT*	Female	547	999	406	2,484	3,146	996	0.464	0.388	1.59×10^{-17}	1.37 (1.27-1.47)
(5p15.33)	intron 2	Male	337	580	289	3,239	3,987	1,303	0.480	0.387	9.98×10^{-36}	1.73 (1.59-1.89)
		Never-smoker	557	973	415	2,870	3,560	1,145	0.463	0.386	1.66×10^{-18}	1.37 (1.28-1.47)
		Smoker	327	606	280	2,853	3,573	1,154	0.481	0.388	5.12×10^{-18}	1.46 (1.34-1.59)
rs2853677-C	*TERT*	Female	748	958	250	3,204	2,789	627	0.373	0.305	2.23×10^{-15}	1.35 (1.25-1.46)
(5p15.33)	intron 2	Male	434	579	194	4,160	3,573	785	0.401	0.302	1.42×10^{-22}	1.55 (1.42-1.69)
		Never-smoker	734	966	248	3,701	3,144	720	0.375	0.303	5.57×10^{-18}	1.38 (1.28-1.49)
		Smoker	448	571	196	3,663	3,218	692	0.396	0.304	9.44×10^{-20}	1.50 (1.38-1.64)
rs2179920-A	*HLA-DPB1*	Female	1,231	627	98	4,673	1,787	165	0.210	0.160	1.57×10^{-13}	1.40 (1.28-1.53)
(6p21.32)	intergenic	Male	748	405	55	5899	2379	250	0.213	0.169	7.46×10^{-8}	1.33 (1.20-1.48)
		Never-smoker	1,220	634	94	5,227	2,140	209	0.211	0.169	8.06×10^{-10}	1.32 (1.21-1.44)
		Smoker	759	398	59	5,345	2,026	206	0.212	0.161	3.37×10^{-10}	1.40 (1.26-1.56)
rs3817963-G	*BTNL2*	Female	769	902	280	3,130	2,843	651	0.375	0.313	4.72×10^{-13}	1.32 (1.22-1.42)
(6p21.3)	intron 4	Male	475	567	166	3,968	3,670	885	0.372	0.319	2.00×10^{-7}	1.26 (1.16-1.38)
		Never-smoker	781	885	280	3,543	3,276	751	0.371	0.316	3.97×10^{-11}	1.28 (1.19-1.38)
		Smoker	463	584	166	3,555	3,237	785	0.378	0.317	3.83×10^{-9}	1.31 (1.19-1.43)
rs7636839-A	*TP63*	Female	475	951	534	1,862	3,268	1,497	0.515	0.472	2.76×10^{-6}	1.19 (1.10-1.27)
(3q28)	intron 1	Male	247	591	371	2,299	4,194	2,031	0.551	0.484	6.95×10^{-10}	1.31 (1.20-1.43)
		Never-smoker	467	930	556	2,081	3,760	1,733	0.523	0.477	3.37×10^{-7}	1.20 (1.12-1.29)
		Smoker	255	612	349	2,080	3,702	1,795	0.539	0.481	1.43×10^{-7}	1.26 (1.16-1.37)
rs7216064-A	*BPTF*	Female	131	724	1,103	595	2,716	3,317	0.748	0.705	1.77×10^{-7}	1.24 (1.14-1.35)
(17q24.2)	intron 9	Male	76	460	675	744	3,512	4,274	0.747	0.707	3.98×10^{-5}	1.23 (1.11-1.35)
		Never-smoker	136	712	1,104	669	3,143	3,766	0.748	0.704	7.75×10^{-8}	1.25 (1.15-1.35)
		Smoker	71	472	674	670	3,085	3,825	0.748	0.708	5.96×10^{-5}	1.22 (1.11-1.35)
rs2495239-A	*FOXP4*	Female	661	938	361	2,513	3,121	992	0.423	0.385	1.67×10^{-5}	1.17 (1.09-1.26)
(6p21.1)	intergenic	Male	412	566	229	3,311	3,962	1,250	0.424	0.379	2.02×10^{-5}	1.21 (1.11-1.32)
		Never-smoker	658	930	363	2,913	3,528	1,131	0.424	0.382	1.57×10^{-6}	1.19 (1.11-1.28)
		Smoker	415	574	227	2,911	3,555	1,111	0.423	0.381	9.68×10^{-5}	1.19 (1.09-1.30)

*A: Protective allele, a: risk allele.

Supplementary Table 7. Association of 7 SNPs with risk for lung adenocarcinoma <u>*WITHOUT*</u> *EGFR* mutation

SNP ID-risk allele	Gene	Stage	Case[a]			HWE[b]	Control[a]			HWE[b]	Risk allele frequency		P_{Trend} value	OR (95%CI)	P_{het}
(Locus)			AA	Aa	aa		AA	Aa	aa		Case	Control			
rs2736100-G	*TERT*	GWAS	208	294	129	0.18	1642	2032	691	0.14	0.437	0.391	1.96×10^{-3}	1.20 (1.07-1.36)[c]	
(5p15.33)	intron 2	1st validation	523	881	344	0.43	2577	3226	1013	0.95	0.449	0.385	2.35×10^{-10}	1.32 (1.21-1.44)[c]	
		2nd validation	442	632	219	0.79	1504	1875	595	0.79	0.414	0.386	5.35×10^{-3}	1.14 (1.04-1.25)[d]	
		All studies combined	1173	1807	692	HWE	5723	7133	2299	0.32	0.435	0.387	4.67×10^{-13}	1.23 (1.16-1.30)[e]	0.073
rs2853677-C	*TERT*	GWAS	248	292	91	0.74	2106	1832	429	0.29	0.376	0.308	1.79×10^{-6}	1.35 (1.19-1.52)[c]	
(5p15.33)	intron 2	1st validation	681	846	221	0.095	3371	2833	611	0.65	0.368	0.298	1.57×10^{-13}	1.40 (1.28-1.53)[c]	
		2nd validation	566	582	166	0.39	1887	1697	372	0.73	0.348	0.309	1.09×10^{-4}	1.21 (1.10-1.33)[d]	
		All studies combined	1495	1720	478	0.63	7364	6362	1412	0.48	0.362	0.303	9.55×10^{-21}	1.32 (1.24-1.39)[e]	0.078
rs2179920-A	*HLA-DPB1*	GWAS	426	181	24	0.39	2969	1271	125	0.43	0.181	0.174	0.51	1.05 (0.90-1.23)[c]	
(6p21.32)	intergenic	1st validation	1141	546	60	0.59	4897	1744	176	0.17	0.191	0.154	5.35×10^{-8}	1.35 (1.21-1.51)[c]	
		2nd validation	891	370	49	0.18	2706	1151	114	0.53	0.179	0.174	0.51	1.04 (0.93-1.17)[d]	
		All studies combined	2458	1097	133	0.44	10572	4166	415	0.85	0.185	0.165	2.21×10^{-5}	1.17 (1.09-1.25)[e]	0.0020
rs3817963-G	*BTNL2*	GWAS	261	302	68	0.16	1985	1906	474	0.61	0.347	0.327	0.15	1.10 (0.97-1.24)[c]	
(6p21.3)	intron 4	1st validation	737	786	224	0.52	3260	2885	664	0.49	0.353	0.309	5.54×10^{-6}	1.23 (1.12-1.34)[c]	
		2nd validation	575	571	169	0.15	1853	1722	398	0.94	0.346	0.317	6.22×10^{-3}	1.14 (1.04-1.26)[d]	
		All studies combined	1573	1659	461	0.47	7098	6513	1536	0.46	0.349	0.316	1.29×10^{-7}	1.17 (1.10-1.24)[e]	0.30
rs7636839-A	*TP63*	GWAS	145	312	174	0.82	1211	2125	1030	0.10	0.523	0.479	4.41×10^{-3}	1.19 (1.05-1.33)[c]	
(3q28)	intron 1	1st validation	432	873	441	1.00	1846	3414	1553	0.74	0.503	0.478	0.013	1.11 (1.02-1.21)[c]	
		2nd validation	316	652	346	0.80	1104	1923	945	0.06	0.511	0.480	8.31×10^{-3}	1.13 (1.03-1.23)[d]	
		All studies combind	893	1837	961	0.80	4161	7462	3528	0.10	0.509	0.479	5.88×10^{-6}	1.13 (1.07-1.20)[e]	0.68
rs7216064-A	*BPTF*	GWAS	35	259	337	0.10	370	1824	2173	0.64	0.739	0.706	0.015	1.18 (1.03-1.35)[c]	
(17q24.2)	intron 9	1st validation	134	664	949	0.24	600	2784	3433	0.29	0.733	0.708	9.93×10^{-4}	1.17 (1.07-1.28)[c]	
		2nd validation	95	541	678	0.36	369	1620	1985	0.14	0.722	0.703	0.071	1.10 (0.99-1.21)[d]	
		All studies combined	264	1464	1964	0.70	1339	6228	7591	0.23	0.730	0.706	1.42×10^{-5}	1.14 (1.08-1.22)[e]	0.56
rs2495239-A	*FOXP4*	GWAS	228	294	109	0.40	1751	1962	653	0.0074	0.406	0.374	0.035	1.14 (1.01-1.28)[c]	
(6p21.1)	intergenic	1st validation	637	827	284	0.57	2595	3216	1006	0.85	0.399	0.383	0.10	1.07 (0.99-1.17)[c]	
		2nd validation	486	614	205	0.63	1478	1905	583	0.44	0.392	0.387	0.43	1.04 (0.95-1.14)[d]	
		All studies combined	1351	1735	598	0.30	5824	7083	2242	0.24	0.398	0.382	0.012	1.07 (1.02-1.14)[e]	0.51

[a]A: Protective allele, a: risk allele. [b]Hardy-Weinberg Equilibrium . [c]Adjusted for age, gender and smoking status. [d]Adjusted for gender and smoking status. [e]Combined meta-analysis was perfomed using a fixed effects model.

Supplementary Table 8. Allele differentiation between lung adenocarcinomas with and without *EGFR* mutaion

SNP ID-risk allele (Locus)	Gene	Stage	LADC with *EGFR* mutation[a]			HWE[b]	LADC without *EGFR* mutation[a]			Risk allele frequency		P_{Trend} value[c]	OR (95%CI)
			AA	Aa	aa		AA	Aa	aa	LADC with EGFR mutation	LADC without EGFR mutation		
rs2736100-G	*TERT*	GWAS	195	316	152	0.27	208	294	129	0.468	0.437	0.23	1.10 (0.94-1.29)
(5p15.33)	intron 2	1st validation	343	638	293	0.91	523	881	344	0.480	0.449	0.028	1.13 (1.01-1.26)
		2nd validation	346	625	250	0.29	442	632	219	0.461	0.414	0.0016	1.21 (1.08-1.37)
		All samples	884	1579	695	0.84	1173	1807	692	0.470	0.435	1.1×10^{-4}	1.15 (1.07-1.24)
rs2853677-C	*TERT*	GWAS	257	312	94	0.96	248	292	91	0.377	0.376	0.96	1.00 (0.84-1.18)
(5p15.33)	intron 2	1st validation	467	627	180	0.19	681	846	221	0.387	0.368	0.18	1.08 (0.96-1.21)
		2nd validation	458	598	170	0.26	566	582	166	0.383	0.348	0.026	1.15 (1.02-1.30)
		All samples	1182	1537	444	0.12	1495	1720	478	0.383	0.362	0.028	1.09 (1.01-1.17)
rs2179920-A	*HLA-DPB1*	GWAS	403	226	34	0.75	426	181	24	0.222	0.181	0.017	1.27 (1.04-1.55)
(6p21.32)	intergenic	1st validation	808	410	54	0.83	1141	546	60	0.204	0.191	0.21	1.09 (0.95-1.25)
		2nd validation	768	396	65	0.14	891	370	49	0.214	0.179	0.0026	1.25 (1.08-1.44)
		All samples	1979	1032	153	0.22	2458	1097	133	0.211	0.185	1.8×10^{-4}	1.18 (1.08-1.29)
rs3817963-G	*BTNL2*	GWAS	251	310	102	0.70	261	302	68	0.388	0.347	0.028	1.21 (1.02-1.43)
(6p21.3)	intron 4	1st validation	493	585	196	0.30	737	786	224	0.383	0.353	0.043	1.12 (1.00-1.25)
		2nd validation	500	574	148	0.39	575	571	169	0.356	0.346	0.38	1.06 (0.94-1.19)
		All samples	1244	1469	446	0.71	1573	1659	461	0.374	0.349	3.6×10^{-3}	1.12 (1.04-1.20)
rs7636839-A	*TP63*	GWAS	146	314	203	0.24	145	312	174	0.543	0.523	0.32	1.09 (0.93-1.27)
(3q28)	intron 1	1st validation	303	617	354	0.29	432	873	441	0.520	0.503	0.11	1.09 (0.98-1.22)
		2nd validation	273	611	348	0.88	316	652	346	0.530	0.511	0.16	1.09 (0.97-1.22)
		All samples	722	1542	905	0.18	893	1837	961	0.529	0.509	0.017	1.09 (1.02-1.16)
rs7216064-A	*BPTF*	GWAS	40	240	383	0.77	35	259	337	0.759	0.739	0.34	1.10 (0.91-1.32)
(17q24.2)	intron 9	1st validation	86	488	701	0.93	134	664	949	0.741	0.733	0.74	1.02 (0.90-1.15)
		2nd validation	81	456	694	0.60	95	541	678	0.749	0.722	0.039	1.15 (1.01-1.31)
		All samples	207	1184	1778	0.60	264	1464	1964	0.748	0.730	0.059	1.08 (0.99-1.17)
rs2495239-A	*FOXP4*	GWAS	207	318	138	0.44	228	294	109	0.448	0.406	0.039	1.18 (1.01-1.39)
(6p21.1)	intergenic	1st validation	450	594	229	0.18	637	827	284	0.413	0.399	0.20	1.07 (0.96-1.20)
		2nd validation	416	592	223	0.62	486	614	205	0.422	0.392	0.10	1.10 (0.98-1.24)
		All samples	1073	1504	590	0.12	1351	1735	598	0.424	0.398	6.1×10^{-3}	1.10 (1.03-1.19)

[a]AA: major homozygote, Aa: heterozygote, aa: minor homozygote. [b]Hardy-Weinberg Equilibrium . [c]Adjusted for age, gender and smoking status.

Supplementary Table 9. SNPs at 6p21.32 (rs2179920) showing association with $P < 1\times10^{-4}$ in GWAS after imputation analysis

SNP ID	Position (hb18)	Gene	Location	Allele/Amino acid		Allele frequency for alt		P_{Trend} value*	RSQR	Effect size	Standard error	Identification by
				Reference	Alteration	Case	Control					
rs2071362	33,141,252	*HLA-DPA1*	intronic	A	G	0.208	0.258	5.6.E-04	1.00	-0.26	0.076	Imputing
rs2301226	33,142,574	*HLA-DPA1*	intronic	G	A	0.208	0.258	5.6.E-04	1.00	-0.26	0.076	Genotyping
rs3135020	33,149,042	*HLA-DPA1*	intronic	C	A	0.043	0.027	8.2.E-04	0.96	0.58	0.166	Imputing
rs2071349	33,151,498	*HLA-DPA1*	intronic	C	G	0.210	0.262	5.3.E-04	1.00	-0.26	0.076	Genotyping
AA_DPB1_57_33156583	33,156,583	*HLA-DPB1*	exonic	E	D	0.219	0.171	6.0.E-04	1.00	0.27	0.078	Imputing
AA_DPB1_84_33156664	33,156,664	*HLA-DPB1*	exonic	D	G	0.357	0.416	4.4.E-05	1.00	-0.27	0.066	Imputing
AA_DPB1_85_33156667	33,156,667	*HLA-DPB1*	exonic	E	G	0.356	0.415	4.1.E-05	1.00	-0.27	0.066	Imputing
AA_DPB1_86_33156670	33,156,670	*HLA-DPB1*	exonic	A	P	0.356	0.416	4.1.E-05	1.00	-0.27	0.066	Imputing
AA_DPB1_87_33156673	33,156,673	*HLA-DPB1*	exonic	V	M	0.356	0.415	4.2.E-05	1.00	-0.27	0.066	Imputing
HLA_DPB1_02	33,157,346	*HLA-DPB1*	allelic	A	P	0.216	0.266	7.8.E-04	0.99	-0.25	0.075	Imputing
rs9277358	33,157,961	*HLA-DPB1*	intronic	A	G	0.362	0.422	2.8.E-05	1.00	-0.27	0.066	Imputing
rs9277361	33,158,023	*HLA-DPB1*	intronic	G	A	0.362	0.422	2.8.E-05	1.00	-0.27	0.066	Imputing
rs9277366	33,158,085	*HLA-DPB1*	intronic	A	G	0.362	0.422	2.8.E-05	1.00	-0.27	0.066	Imputing
rs9277373	33,158,157	*HLA-DPB1*	intronic	G	A	0.362	0.422	2.8.E-05	1.00	-0.27	0.066	Imputing
rs6899657	33,158,201	*HLA-DPB1*	intronic	A	G	0.381	0.444	1.3.E-05	1.00	-0.28	0.066	Imputing
rs9277378	33,158,257	*HLA-DPB1*	intronic	G	A	0.362	0.422	2.8.E-05	1.00	-0.27	0.066	Genotyping
rs9277379	33,158,303	*HLA-DPB1*	intronic	A	C	0.221	0.171	3.5.E-04	1.00	0.28	0.078	Imputing
rs9277384	33,158,441	*HLA-DPB1*	intronic	C	G	0.221	0.171	3.5.E-04	1.00	0.28	0.078	Imputing
rs9277385	33,158,451	*HLA-DPB1*	intronic	G	A	0.362	0.422	2.8.E-05	1.00	-0.27	0.066	Imputing
rs9277386	33,158,477	*HLA-DPB1*	intronic	G	A	0.323	0.375	1.4.E-04	1.00	-0.25	0.067	Genotyping
rs9277389	33,158,504	*HLA-DPB1*	intronic	A	G	0.362	0.422	2.8.E-05	1.00	-0.27	0.066	Imputing
rs3097674	33,158,661	*HLA-DPB1*	intronic	G	A	0.362	0.422	2.8.E-05	1.00	-0.27	0.066	Imputing
rs9277393	33,158,855	*HLA-DPB1*	intronic	G	A	0.357	0.418	2.2.E-05	1.00	-0.28	0.066	Imputing
rs9277396	33,159,117	*HLA-DPB1*	intronic	A	G	0.357	0.418	2.2.E-05	1.00	-0.28	0.066	Genotyping
rs9277421	33,159,798	*HLA-DPB1*	intronic	A	G	0.357	0.418	2.2.E-05	1.00	-0.28	0.066	Imputing
rs9277426	33,159,888	*HLA-DPB1*	intronic	A	C	0.357	0.418	2.2.E-05	1.00	-0.28	0.066	Genotyping
rs9277437	33,160,228	*HLA-DPB1*	intronic	G	A	0.357	0.418	2.2.E-05	1.00	-0.28	0.066	Imputing
AA_DPB1_96_33160714_K	33,160,714	*HLA-DPB1*	exonic	P	A	0.360	0.419	3.4.E-05	1.00	-0.27	0.066	Imputing
AA_DPB1_96_33160714_R	33,160,714	*HLA-DPB1*	exonic	A	P	0.355	0.416	1.8.E-05	1.00	-0.28	0.066	Imputing
AA_DPB1_170_33160936_I	33,160,936	*HLA-DPB1*	exonic	P	A	0.360	0.419	3.4.E-05	1.00	-0.27	0.066	Imputing
AA_DPB1_170_33160936_T	33,160,936	*HLA-DPB1*	exonic	A	P	0.355	0.416	1.8.E-05	1.00	-0.28	0.066	Imputing
rs9277462	33,161,249	*HLA-DPB1*	intronic	G	A	0.357	0.418	2.2.E-05	1.00	-0.28	0.066	Imputing
rs9277464	33,161,330	*HLA-DPB1*	intronic	A	G	0.357	0.418	2.2.E-05	1.00	-0.28	0.066	Genotyping
rs9277469	33,161,446	*HLA-DPB1*	intronic	A	C	0.357	0.418	2.2.E-05	1.00	-0.28	0.066	Genotyping
rs9277471	33,161,660	*HLA-DPB1*	intronic	A	G	0.357	0.418	2.2.E-05	1.00	-0.28	0.066	Genotyping
rs9277472	33,161,701	*HLA-DPB1*	intronic	G	A	0.357	0.418	2.2.E-05	1.00	-0.28	0.066	Imputing
rs9277489	33,161,920	*HLA-DPB1*	intronic	G	A	0.357	0.418	2.2.E-05	1.00	-0.28	0.066	Imputing
rs1042544	33,162,435	*HLA-DPB1*	3'-UTR	G	A	0.358	0.419	2.1.E-05	1.00	-0.28	0.066	Imputing
rs931	33,162,528	*HLA-DPB1*	3'-UTR	A	G	0.357	0.418	2.2.E-05	1.00	-0.28	0.066	Imputing
rs9277533	33,162,699	*HLA-DPB1*	3'-UTR	A	G	0.357	0.418	2.2.E-05	1.00	-0.28	0.066	Genotyping
rs9277535	33,162,839	*HLA-DPB1*	3'-UTR	G	A	0.357	0.418	2.2.E-05	1.00	-0.28	0.066	Imputing
rs9277538	33,163,025	*HLA-DPB1*	3'-UTR	G	A	0.357	0.418	2.2.E-05	1.00	-0.28	0.066	Imputing
rs9277542	33,163,225	*HLA-DPB1*	3'-UTR	G	A	0.357	0.418	2.2.E-05	1.00	-0.28	0.066	Imputing
rs9277545	33,163,301	*HLA-DPB1*	3'-UTR	A	G	0.376	0.440	1.0.E-05	1.00	-0.29	0.065	Imputing
rs9277546	33,163,324	*HLA-DPB1*	3'-UTR	C	A	0.357	0.418	2.2.E-05	1.00	-0.28	0.066	Genotyping
rs9277554	33,163,516	*HLA-DPB1*	3'-UTR	A	G	0.357	0.418	2.2.E-05	1.00	-0.28	0.066	Genotyping
rs9277555	33,163,583	*HLA-DPB1*	3'-UTR	A	G	0.376	0.440	9.8.E-06	1.00	-0.29	0.065	Genotyping
rs3128965	33,163,877	*HLA-DPB1*	3'-UTR	G	A	0.221	0.171	2.8.E-04	1.00	0.29	0.078	Imputing
rs3117229	33,164,047	*HLA-DPB1*	3'-UTR	A	G	0.357	0.418	2.2.E-05	1.00	-0.28	0.066	Genotyping
rs3130186	33,164,185	*HLA-DPB1*	3'-UTR	A	G	0.357	0.418	2.2.E-05	1.00	-0.28	0.066	Imputing
rs3117228	33,164,413	*HLA-DPB1*	3'-UTR	A	C	0.357	0.418	2.2.E-05	1.00	-0.28	0.066	Genotyping
rs3091281	33,164,544	*HLA-DPB1*	3'-UTR	G	A	0.357	0.418	2.3.E-05	1.00	-0.28	0.066	Imputing
rs9277557	33,164,672	*HLA-DPB1*	3'-UTR	G	A	0.357	0.418	2.2.E-05	1.00	-0.28	0.066	Genotyping
rs9277561	33,164,727	*HLA-DPB1*	3'-UTR	G	A	0.376	0.440	1.0.E-05	1.00	-0.29	0.066	Imputing
rs9277565	33,164,875	*HLA-DPB1*	3'-UTR	A	G	0.376	0.440	9.3.E-06	1.00	-0.29	0.065	Genotyping
rs9277567	33,164,991	*HLA-DPB1*	3'-UTR	C	A	0.376	0.440	1.0.E-05	1.00	-0.29	0.065	Imputing
rs3130188	33,165,154	*HLA-DPB1*	3'-UTR	G	A	0.357	0.418	2.2.E-05	1.00	-0.28	0.066	Genotyping
rs3117226	33,165,637	*HLA-DPB1*	intergenic	A	G	0.381	0.444	1.2.E-05	1.00	-0.28	0.066	Genotyping
rs3117225	33,165,689	*HLA-DPB1*	intergenic	A	G	0.357	0.419	1.9.E-05	1.00	-0.28	0.066	Genotyping
rs3097652	33,165,813	*HLA-DPB1*	intergenic	A	G	0.357	0.418	2.2.E-05	1.00	-0.28	0.066	Genotyping
rs1367730	33,166,092	*HLA-DPB1*	intergenic	A	G	0.376	0.440	9.8.E-06	1.00	-0.29	0.065	Genotyping
rs3128972	33,166,752	*HLA-DPB1*	intergenic	G	A	0.376	0.440	9.8.E-06	1.00	-0.29	0.065	Genotyping
rs2179920	33,166,852	*HLA-DPB1*	intergenic	G	A	0.222	0.172	2.6.E-04	1.00	0.29	0.078	Genotyping
rs2179919	33,167,240	*HLA-DPB1*	intergenic	G	A	0.376	0.440	1.0.E-05	1.00	-0.29	0.065	Genotyping
rs2281389	33,167,774	*HLA-DPB1*	intergenic	A	G	0.222	0.172	3.1.E-04	1.00	0.28	0.078	Genotyping
rs3128917	33,167,974	*HLA-DPB1*	intergenic	C	A	0.376	0.440	1.2.E-05	1.00	-0.28	0.065	Genotyping
rs3117222	33,168,927	*HLA-DPB1*	intergenic	A	G	0.376	0.440	9.3.E-06	1.00	-0.29	0.065	Genotyping
rs3130190	33,169,668	*HLA-DPB1*	intergenic	G	A	0.377	0.440	1.3.E-05	1.00	-0.28	0.065	Genotyping
rs3130191	33,169,849	*HLA-DPB1*	intergenic	G	A	0.377	0.440	1.3.E-05	1.00	-0.28	0.065	Genotyping
rs3117221	33,169,925	*HLA-DPB1*	intergenic	A	G	0.377	0.440	1.3.E-05	1.00	-0.28	0.065	Genotyping
rs3130198	33,171,909	*HLA-DPB1*	intergenic	G	A	0.377	0.440	1.3.E-05	1.00	-0.28	0.065	Genotyping
rs3117213	33,172,583	*HLA-DPB1*	intergenic	A	C	0.377	0.440	1.3.E-05	1.00	-0.28	0.065	Genotyping
rs2395319	33,175,189	*HLA-DPB1*	intergenic	A	G	0.377	0.440	1.3.E-05	1.00	-0.28	0.065	Genotyping
rs3117239	33,179,755	*HLA-DPB1*	intergenic	A	G	0.221	0.172	3.2.E-04	1.00	0.28	0.078	Genotyping
rs2064479	33,180,218	*HLA-DPB1*	intergenic	A	G	0.377	0.440	1.3.E-05	1.00	-0.28	0.065	Genotyping
rs2064478	33,180,244	*HLA-DPB1*	intergenic	G	A	0.221	0.172	3.2.E-04	1.00	0.28	0.078	Genotyping
rs3130210	33,180,707	*HLA-DPB1*	intergenic	C	A	0.221	0.171	3.7.E-04	1.00	0.28	0.078	Imputing
rs2064476	33,181,300	*HLA-DPB1*	intergenic	G	A	0.358	0.418	3.0.E-05	1.00	-0.27	0.066	Genotyping
rs3117234	33,181,962	*HLA-DPB1*	intergenic	A	G	0.221	0.172	3.2.E-04	1.00	0.28	0.078	Genotyping
rs3130212	33,182,367	*HLA-DPB1*	intergenic	G	C	0.220	0.171	3.5.E-04	1.00	0.28	0.078	Imputing
rs3117231	33,182,886	*HLA-DPB1*	intergenic	G	A	0.382	0.444	1.7.E-05	1.00	-0.28	0.065	Genotyping
rs3117230	33,183,613	*HLA-DPB1*	intergenic	A	G	0.221	0.172	3.2.E-04	1.00	0.28	0.078	Genotyping
rs9277628	33,190,018	*HLA-DPB2*	intronic	A	C	0.217	0.266	9.3.E-04	0.99	-0.24	0.075	Imputing
rs9277935	33,268,403	*COL11A2*	intergenic	A	C	0.305	0.348	4.5.E-04	1.00	-0.24	0.070	Genotyping
rs2076310	33,274,012	*RXRB*	intronic	G	A	0.302	0.345	6.7.E-04	1.00	-0.24	0.070	Genotyping
rs439205	33,281,820	*HSD17B8*	intronic	A	G	0.302	0.345	5.3.E-04	1.00	-0.24	0.070	Genotyping
rs421446	33,282,761	*HSD17B8*	intergenic	G	A	0.302	0.345	5.3.E-04	1.00	-0.24	0.070	Genotyping
rs107822	33,283,553	*HSD17B8*	intergenic	A	G	0.302	0.345	5.1.E-04	1.00	-0.24	0.070	Genotyping
rs213208	33,285,988	*RING1*	intronic	A	C	0.302	0.346	5.2.E-04	1.00	-0.24	0.070	Genotyping
rs2854028	33,287,667	*RING1*	synonymous	G	A	0.143	0.183	5.9.E-04	1.00	-0.30	0.089	Genotyping
rs386084	33,300,640	*RING1*	intergenic	A	G	0.314	0.355	9.7.E-04	1.00	-0.23	0.069	Genotyping

*Adjusted for age, gender and smoking status.

Supplementary Table 10. SNPs at 6p21.32 (rs3817963) showing association with $P_{\text{Trend}} < 1×10^{-4}$ in GWAS after imputation analysis

SNP ID	Position (hb18)	Gene	Location	Allele		Allele frequency for alt		P_{Trend} value*	RSQR	Effect size	Standard error	Identification by
				Reference	Alteration	Case	Control					
rs3817971	32,469,111	*BTNL2*	downstream	A	G	0.300	0.250	8.8.E-04	0.87	0.24	0.072	Imputing
rs3817968	32,469,428	*BTNL2*	downstream	A	G	0.300	0.250	8.6.E-04	0.87	0.24	0.072	Imputing
rs3817967	32,469,447	*BTNL2*	downstream	G	A	0.300	0.250	8.5.E-04	0.87	0.24	0.072	Imputing
rs2076531	32,471,690	*BTNL2*	intronic	A	G	0.278	0.224	3.3.E-04	1.00	0.26	0.071	Imputing
rs2076528	32,472,172	*BTNL2*	intronic	A	C	0.278	0.224	3.3.E-04	1.00	0.26	0.071	Genotyping
rs2076527	32,472,192	*BTNL2*	intronic	A	G	0.278	0.224	3.3.E-04	1.00	0.26	0.071	Genotyping
rs3817966	32,475,825	*BTNL2*	intronic	A	G	0.387	0.324	5.9.E-05	1.00	0.26	0.065	Imputing
rs3817963	32,476,065	*BTNL2*	intronic	A	G	0.388	0.323	4.9.E-05	1.00	0.26	0.065	Genotyping
rs3763315	32,484,632	*BTNL2*	upstream	C	A	0.278	0.224	3.5.E-04	1.00	0.26	0.071	Genotyping
rs9380293	32,485,262	*BTNL2*	upstream	G	A	0.306	0.252	6.2.E-04	1.00	0.24	0.069	Genotyping
rs9405098	32,487,714	*BTNL2*	upstream	G	A	0.306	0.252	6.2.E-04	1.00	0.24	0.069	Genotyping

*Adjusted for age, gender and smoking status.

Supplementary Table 11. Association results of SNPs at 6p21.32 in conditional analysis

Stage	SNP ID	Gene	Location	Allele		Conditioned on rs3817963				Conditioned on rs2179920			
				Reference	Alteration	OR	95% CI		P_{Trend} value	OR	95% CI		P_{Trend} value
							Lower	Upper			Lower	Upper	
GWAS	rs3817963	*BTNL2*	intronic	A	G	—	—	—	1	1.23[a]	1.07	1.41	3.5.E-03
	rs2179920	*HLA-DPB1*	intergenic	C	T	1.22[a]	1.03	1.43	2.1.E-02	—	—	—	1
Validation set 1	rs3817963	*BTNL2*	intronic	A	G	—	—	—	1	1.28[a]	1.15	1.43	4.4.E-06
	rs2179920	*HLA-DPB1*	intergenic	C	T	1.29[a]	1.13	1.47	1.2.E-04	—	—	—	1
Validation set 2	rs3817963	*BTNL2*	intronic	T	C	—	—	—	1	1.12[b]	1.00	1.24	0.0429
	rs2179920	*HLA-DPB1*	intergenic	C	T	1.25[b]	1.10	1.42	4.4.E-04	—	—	—	1
Combined all studies	rs3817963	*BTNL2*	intronic	A	G	—	—	—	1	1.20[c]	1.13	1.28	3.46E-08
	rs2179920	*HLA-DPB1*	intergenic	C	T	1.26[c]	1.16	1.36	1.34E-08	—	—	—	1

[a]Adjusted for age, gender and smoking status. [b]Adjusted for gender and smoking status. [c]Combined meta-analysis was perfomed using a fixed effects model.

Supplementary Table 12. Linkage disequilibrium among SNPs in the HLA-class II region

SNP ID/allele	Position	R^2 value between two SNPs			
		rs3817963	DPB1_the 57 residue	rs2179920	rs2495239
rs3817963	32,476,065	-	0.150	0.144	0.00
DPB1_the 57 residue	33,156,583	0.150	-	0.977	0.00
rs2179920	33,166,852	0.144	0.977	-	0.00
rs2495239	41,522,750	0.00	0.00	0.00	-

Supplementary Table 13. Differences in the distribution of the *HLA-DPB1* alleles between cases and controls in GWAS.

4-digit HLA alleles (HLA-DPB1)	Japanese			East Asian[4]	European[5]	Amino acid position in HLA-DPB1					Minor allele frequency		P_{Trend} value	Effect size	Standard error
	Japanese reference panel (N=908)[1]	HLA Laboratory (N=2,966)[2]	JPDSC (N=2,823)[3]			8	9	11	**57**	76	Case	Control			
*02:01	0.226	0.241	0.233	0.164	0.145	L	F	G	E	M	0.185	0.228	4.2.E-03	-0.224	0.079
*02:02	0.042	0.034	0.039	0.049	0.019	L	F	G	E	M	0.031	0.039	1.2.E-01	-0.272	0.181
*03:01	0.034	0.040	0.048	0.074	0.116	V	Y	L	**D**	V	0.065	0.047	1.6.E-02	0.321	0.130
*04:01	0.046	0.051	0.059	0.197	0.373	L	F	G	E	M	0.038	0.049	2.0.E-02	-0.359	0.159
*04:02	0.093	0.098	0.097	0.024	0.091	L	F	G	E	M	0.100	0.098	8.5.E-01	-0.020	0.103
*05:01	0.389	0.384	0.377	0.204	0.030	L	F	G	E	M	0.403	0.389	1.2.E-01	0.100	0.065
*06:01	0.004	0.006	0.006	0.000	0.016	V	Y	L	**D**	M	0.011	0.007	1.1.E-01	0.545	0.329
*09:01	0.126	0.099	0.100	0.029	0.008	V	H	L	**D**	V	0.129	0.101	1.5.E-02	0.241	0.098
*13:01	0.016	0.020	0.017	0.099	0.028	V	Y	L	E	I	0.014	0.017	3.6.E-01	-0.232	0.257
*14:01	0.013	0.015	0.014	0.029	0.015	V	H	L	**D**	V	0.013	0.015	3.6.E-01	-0.246	0.274

[1]Okada et al., Nat. Genet. 47, 798–802 (2015) , [2]http://hla.or.jp/med/frequency_search/en/haplo/, [3]Kamitsuji et al., J Hum Genet. 60, 319-326 (2015), [4]East Asian are not inclued the HapMap JPT, [5]European population are the HapMap CEU.

Supplementary Table 14. SNPs at 6p21.1 (rs2495239) showing associaition with $P < 1\times10^{-4}$ in GWAS after imputation analysis

SNP ID	Position	Gene	Location	Allele		Frequency of the reference allele		P_{Trend} value*	RSQR	Effect size	Standard error	Identification by
				Reference	Alteration	Case	Control					
rs2894439	41,480,093	*LINC01276*	ncRNA_intronic	G	A	0.560	0.636	8.01E-09	0.86	-0.378	0.065	Imputing
rs181350472	41,483,123	*LINC01276*	ncRNA_intronic	A	G	0.616	0.684	3.20E-08	0.79	-0.390	0.070	Imputing
rs9367106	41,483,390	*LINC01276*	ncRNA_intronic	G	C	0.616	0.684	3.20E-08	0.79	-0.389	0.070	Imputing
rs9367107	41,483,450	*LINC01276*	ncRNA_intronic	G	A	0.615	0.683	3.32E-08	0.80	-0.387	0.070	Imputing
rs1886818	41,484,798	*LINC01276*	ncRNA_intronic	T	C	0.630	0.562	2.58E-06	0.98	0.294	0.063	Imputing
rs1886817	41,484,959	*LINC01276*	ncRNA_intronic	T	A	0.630	0.562	2.56E-06	0.98	0.294	0.063	Imputing
rs2477839	41,485,594	*LINC01276*	ncRNA_intronic	C	T	0.654	0.585	1.42E-06	0.99	0.305	0.063	Imputing
rs2477840	41,486,798	*LINC01276*	ncRNA_intronic	C	T	0.654	0.585	1.40E-06	0.99	0.305	0.063	Imputing
rs2496647	41,486,990	*LINC01276*	ncRNA_intronic	G	A	0.659	0.592	2.03E-06	0.98	0.302	0.064	Imputing
rs2495236	41,488,200	*LINC01276*	downstream	G	C	0.654	0.585	1.38E-06	0.99	0.304	0.063	Imputing
rs12660421	41,488,378	*LINC01276*	downstream	G	A	0.638	0.710	5.42E-09	0.82	-0.409	0.070	Imputing
rs2477841	41,488,935	*FOXP4*	intergenic	C	G	0.568	0.501	2.04E-07	0.85	0.347	0.067	Imputing
rs2477842	41,489,441	*FOXP4*	intergenic	C	T	0.654	0.585	1.30E-06	1.00	0.304	0.063	Genotyping
rs2495239	41,490,238	*FOXP4*	intergenic	A	G	0.448	0.374	8.40E-07	1.00	-0.296	0.060	Genotyping
rs41435745	41,490,382	*FOXP4*	intergenic	G	C	0.638	0.710	6.60E-09	0.82	-0.405	0.070	Imputing
rs191773431	41,492,696	*FOXP4*	intergenic	A	T	0.654	0.586	1.66E-06	0.99	0.303	0.063	Imputing
rs7741164	41,493,412	*FOXP4*	intergenic	G	A	0.639	0.710	7.33E-09	0.82	-0.406	0.070	Imputing
rs4714472	41,493,446	*FOXP4*	intergenic	G	A	0.587	0.529	1.54E-05	0.81	0.295	0.068	Imputing
rs1853837	41,497,035	*FOXP4*	intergenic	C	A	0.662	0.722	2.89E-07	0.77	-0.376	0.073	Imputing
rs55889968	41,501,225	*FOXP4*	intergenic	A	G	0.665	0.715	1.73E-05	0.81	-0.308	0.072	Imputing
rs12175265	41,501,834	*FOXP4*	intergenic	G	A	0.665	0.715	1.88E-05	0.81	-0.306	0.072	Imputing
rs1886814	41,502,683	*FOXP4*	intergenic	A	C	0.663	0.712	2.56E-05	0.81	-0.302	0.072	Imputing
rs4714474	41,503,561	*FOXP4*	intergenic	G	A	0.666	0.715	2.59E-05	0.82	-0.300	0.071	Imputing
rs9381074	41,505,196	*FOXP4*	intergenic	T	A	0.664	0.710	2.56E-05	0.72	-0.319	0.076	Imputing

*Adjusted for age, gender and smoking status.

Supplementary Table 15. Association of rs7741164 SNP with risk for lung adenocarcinoma with *EGFR* mutation

SNP ID-risk allele[a] (Locus)	Gene	Stage	Risk allele frequency		P_{Trend} value	OR (95%CI)	95% Confidence interval		P_{het}
			Case	Control			Lower	Upper	
rs7741164-A	*FOXP4*	GWAS[b]	0.361	0.290	7.33×10^{-9}	1.50	1.31	1.72	0.0093
(6p21.1)	intergenic	1st validation[b]	0.336	0.287	5.80×10^{-6}	1.26	1.14	1.39	
		2nd validation[c]	0.336	0.302	4.44×10^{-3}	1.15	1.05	1.27	
		All studies combined[d]	-	-	3.33×10^{-13}	1.26	1.18	1.34	

rs7741164 SNP were perfomed the imputation analyses for GWAS all samples and JPDSC controls in validation set 2 (see "Analysis of the association between the rs7741164 SNP and susceptibility to EGFR mutation-positive LADC" in the online methods). Other subjects, 6,817 controls and 2,510 cases, were genotyped using the invader assay or TaqMan method. [a]A: Protective allele, a: risk allele. [b]Adjusted for age, gender and smoking status. [c]Adjusted for gender and smoking status. [d]Combined meta-analysis was perfomed using a fixed effects model.

Supplementary Table 16. Correlation between genotypes for rs2495239 and the expression of *FOXP4* in non-cancerous lung tissues from 403 individuals

Genotype	Number	Levels of mRNA expression	
		Median $\pm$ SD	*P* value[a]
GG	146	0.833 $\pm$ 0.025	0.023
GA	182	0.850 $\pm$ 0.023	
AA	75	0.948 $\pm$ 0.035	

[a]*P* value was calculated using a multivariate linear regression model. Variables used for adjustment in each test were age (continuous), gender (male or female) and smoking status (never or ever).

Supplementary Table 17. Risk allele frequencies of seven SNPs accroding to ethnicity

SNP ID	Location	Chr	Position (hg38)	Gene	Allele		Allele frequency[a]				Frequency[b]	
					Ref	Risk	AFR	AMR	EAS	EUR	Case	Ctrl
rs2736100	5p15.33	5	1,286,401	*TERT*	T	G	0.43	0.45	0.39	0.50	0.470	0.387
rs2853677	5p15.33	5	1,287,079	*TERT*	T	C	0.29	0.35	0.33	0.40	0.383	0.303
rs3817963	6p21.3	6	32,400,310	*BTNL2*	A	G	0.11	0.31	0.23	0.27	0.374	0.316
rs2179920	6p21.32	6	33,091,097	*HLA-DPB1*	A	G	0.48	0.19	0.14	0.23	0.211	0.165
rs7636839	3q28	3	189,639,152	*TP63*	G	A	0.67	0.62	0.47	0.51	0.529	0.479
rs7216064	17q24.3	17	67,902,693	*BPTF*	A	G	0.83	0.74	0.35	0.78	0.748	0.706
rs2495239	6p22.1	6	41,522,750	*FOXP4*	G	A	0.30	0.22	0.41	0.08	0.424	0.382

Chr, Chromosome; AFR, African; AMR, Admixed American; EAS, East Asian; EUR, European.

[a]Allele frequency among 1000 genomes. [b]Allele frequency in the present study.
